# Supplementary material for: Mangiferin Ameliorates Obesity-Associated Inflammation and Autophagy in High-Fat-Diet-Fed Mice: In Silico and In Vivo Approaches
Source: Int J Mol Sci. 2022 Dec 5;23(23):15329. doi: 10.3390/ijms232315329 (PMC9735994; doi:10.3390/ijms232315329)
Supplement: Supplementary file 1 [file ijms-23-15329-s001.zip › ijms-2014135-supplementary.pdf]

Supplementary Materials

Mangiferin Ameliorates Obesity-Associated Inflammation and Autophagy in High-Fat-Diet-Fed Mice: In Silico and In Vivo Approaches

Ji-Won Noh, Han-Young Lee and Byung-Cheol Lee *

Department of Clinical Korean Medicine, Graduate School, Kyung Hee University, 26 Kyungheedae-to, Dongdaemun-gu, Seoul 02447, Republic of Korea

* Correspondence: hydrolee@khu.ac.kr; Tel.: +82-2-958-9151; Fax: +82-2-958-9182

Supplementary Tables

Table S1. Composition of High Fat Diets (D12492).

Description	Ingredients	Grams
Fat	Lard	245.0
Fat	Soybean Oil, USP	25.0
Protein	Casein, Lactic, 30 mesh	200.0
Protein	Cystine, L	3.0
Carbohydrate	Lodex 10	125.0
Carbohydrate	Sucrose, Fine Granulated	72.8
Fiber	Solka Floc, FCC200	50.0
Mineral	Mineral mix	50.0
Vitamin	Choline Vitartrate	2.0
	Vitamin mix	1.0
		Total: 773.8
